# Single-Shot Multi-Frame Imaging of Cylindrical Shock Waves in a Multi-Layered Assembly

**DOI:** 10.1038/s41598-019-40037-3

**Published:** 2019-03-06

**Authors:** Leora Dresselhaus-Cooper, Joshua E. Gorfain, Chris T. Key, Benjamin K. Ofori-Okai, Suzanne J. Ali, Dmitro J. Martynowych, Arianna Gleason, Steven Kooi, Keith A. Nelson

**Affiliations:** 1 Lawrence Livermore National Laboratory, 7000 East Ave, L-487, Livermore, CA 94550 USA; 2 Department of Chemistry, Massachusetts Institute of Technology, 77 Massachusetts Avenue, Cambridge, MA 02139 USA; 3 Institute for Soldier Nanotechnology, Massachusetts Institute of Technology, 500 Technology Square, NE47-598, Cambridge, MA 02139 USA; 4 Applied Physical Sciences, 4301 North Fairfax Dr., Suite 640, Arlington, VA 22203 USA; 5 SLAC National Accelerator, 2575 Sand Hill Rd, Menlo Park, CA 94025 USA

## Abstract

We demonstrate single-shot multi-frame imaging of quasi-2D cylindrically converging shock waves as they propagate through a multi-layer target sample assembly. We visualize the shock with sequences of up to 16 images, using a Fabry-Perot cavity to generate a pulse train that can be used in various imaging configurations. We employ multi-frame shadowgraph and dark-field imaging to measure the amplitude and phase of the light transmitted through the shocked target. Single-shot multi-frame imaging tracks geometric distortion and additional features in our images that were not previously resolvable in this experimental geometry. Analysis of our images, in combination with simulations, shows that the additional image features are formed by a coupled wave structure resulting from interface effects in our targets. This technique presents a new capability for tabletop imaging of shock waves that can be extended to experiments at large-scale facilities.

## Introduction

The destructive and variable nature of shock waves places high importance on techniques that can provide time-dependent observations in a single experiment^1,2^. Multi-frame imaging of shock waves generated by projectile or explosive impact has been demonstrated on length scales of microns through many meters and time scales of nanoseconds through seconds^3,4^. Laser-generated shock waves are typically monitored optically, including by imaging; however in most cases the shock wave propagates toward the observer^1,5^. The temporal resolution of measurements that image the shock is set by the relationship between the shock velocity and the imaging resolution^6,7^. Imaging methods can also record spatial variation in the shock profile by measuring the side-on spatial profile of the shock^7^. Viewing the waves perpendicular to their direction of propagation allows for monitoring of the spatiotemporal evolution of shock waves as they move through a material.

Pezeril *et*. *al*.^6^ developed a method for laser generation of converging shock waves in a layered sample assembly consisting of a thin (~10 μm) liquid layer sandwiched between two glass substrates. The shock wave was launched by a laser pulse that was focused to a 200-μm diameter circular “ring” pattern at the sample. Absorption of the light in the liquid layer (India ink, i.e. water with amorphous carbon) initiated quasi-2D shock propagation and focusing which increased the pressure of the laser-induced shocks by an order of magnitude, enabling this tabletop experiment to reach high pressures that are usually only attainable in flyer plate, gas gun, or high energy laser facilities^8,9^. This table-top approach enables us to develop new diagnostic techniques to study converging shock waves and the high-pressure dynamics they induce. The geometric instability of converging shock waves makes it difficult to track their propagation with conventional diagnostics that disrupt the wave geometry and thus its properties^8^. As experiments on converging shock waves typically require specialized ultrahigh-energy laser facilities^8,9^, detonation chambers^10^, or high-current facilities^11^, diagnostic development is challenging because of the limited facility time allocated to each experiment. In our case shock propagation in the sample plane, perpendicular to the direction of the light beams, can be monitored by imaging with simultaneously high spatial and temporal resolution. The shock profile can be extracted using interferometric imaging and an empirical formula relating the refractive index to the density^12^. Previous work gathered sequences of images showing the shock’s trajectory by assembling single-frame measurements with varied delays between the excitation and imaging pulses from different regions of the sample.

While this technique proved informative for studying low-pressure, reproducible shock waves in samples that were essentially uniform spatially, challenges remained in characterizing the dynamics of events that differ from shot to shot, such as geometric instabilities near the center of convergence^13^, fracture^14^, and shock-induced decomposition of energetic materials^15^. To measure these and other phenomena whose details are not reproducible, we need to collect the entire image sequence in a single shock event.

In this paper, we present an extension of the capabilities of our previously reported technique^6,12,16^ in which we use a multi-frame imaging method to record dynamical information in a single shot. A Fabry-Perot cavity produces a sequence of ultrafast imaging pulses with a temporal separation that is adjusted to match the timing of a sequence of electronically gated CCDs in a high-frame-rate multi-frame camera. We demonstrate the utility of this multi-frame imaging technique in both shadowgraph and dark-field modes to acquire image sequences showing the progression of the shock wave through our targets. The image sequences capture geometrical distortions in the wave and high-order interface interactions that vary on a shot-to-shot basis due to fluctuations in the drive beam and small variations in the sample. These image sequences show multiple converging and diverging shock features which we compared to hydrodynamic simulations, revealing a coupled-wave structure caused by the interfaces in the targets. Thus single-shot multi-frame imaging has revealed important features that single-frame images could not discern about shock propagation in our multi-layer targets, including features such as geometric instability as well as coupled-wave effects at interfaces that have rarely been observed directly in any sample geometry.

## Experimental Methods

**Figure 1:**
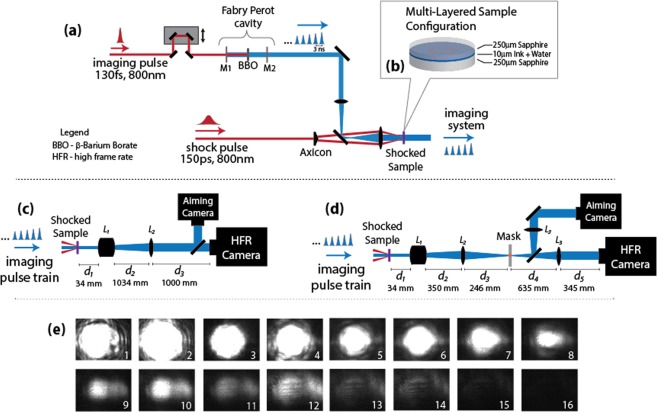
Schematic representations of (**a**) the optical configuration for shock generation, with a Fabry-Perot cavity to create a train of ultrafast imaging pulses for multi-frame imaging diagnostics. (**b**) The multi-layered target configuration used for this work, with a depiction of the drive laser excitation “ring” pattern and expected shock behavior. The optical configurations for (**c**) shadowgraph, and (**d**) dark-field imaging of the shocked sample. (**e**) Image sequence showing the decreasing illumination of each successive imaging pulse output from the Fabry-Perot cavity. The pulses were apertured with an iris after the cavity, and detected with CCDs using no electronic gain. All subsequent image sequences use the detector gain to standardize the image brightness between frames.

The experimental setup is illustrated in Fig. 1. We used a multi-stage amplified Ti:Sapphire laser system, with the oscillator output stretched to 150 ps and amplified at a 10 Hz repetition rate in two stages, first to 4 mJ in a regenerative amplifier and then to 30 mJ in a six-pass bowtie amplifier. We used a half-wave plate and polarizer as a variable beamsplitter to separate 4 mJ of the amplified uncompressed pulse for use as the drive pulse. The rest was compressed to 130 fs and used for the imaging probe.

The drive pulse was shaped into a 150 μm inner diameter ring (8 μm ring width) by passing the 800 nm uncompressed pulse through a 0.5° axicon (a conical prism) and an *f* = 30 mm lens. The femtosecond probe pulse was passed into a frequency-doubling Fabry-Perot cavity, and the output sequence of probe pulses was directed through the target and imaged onto single- and multi-frame cameras. We positioned the sample within the excitation ring pattern using the single-frame camera. A Specialized Imaging SIM 16X multi-frame camera collected up to 16 images with as short as 3 ns inter-frame intervals on electronically gated intensified CCDs.

The target assembly had a ~10-μm thick layer of 10 vol% India Ink (an aqueous solution of amorphous carbon nanoclusters; Winsor & Newton, 951 Black Indian Ink) in deionized water placed between two 250 μm thick, 25.4 mm diameter *r*-cut (i.e. sapphire cut along the ($1\bar{1}02$) face) sapphire wafers. Interaction of the intense drive light by the amorphous carbon in the ink launched high-amplitude expansion waves that formed a shock^6^. All the experiments were conducted using a drive laser pulse energy of 1.0 mJ, corresponding to a fluence of 26.5 J/cm^2^ and an intensity of 1.70 × 10^11^ W/cm^2^, to generate the shock waves. Within the sample plane, expansion of the carbon nanoclusters formed two shock waves in water – one diverging outward, and the other converging inward toward the center of the ring. Additional features are described in the Results and Simulations sections.

The essential components of our imaging setups are shown schematically in Fig. 1. For each imaging system, ~10 mJ of compressed light was directed from the laser into the probe beam. The probe was passed through an adjustable optical delay and then into a frequency-doubling Fabry-Perot cavity to generate a pulse train (Fig. 1a). The cavity consisted of a pair of mirrors, M1 and M2, which were highly transmissive (T_800_ > 99%) at 800 nm wavelength, but reflective to 400 nm wavelength (R_400_ > 99% for M1 and R_400_ = 93% for M2). After coupling into the cavity through M1, some of the 800 nm pulse was frequency-doubled to 400 nm light using a β-barium borate (BBO) crystal with anti-reflection coatings for both 400 nm and 800 nm. The unconverted 800 nm light exited the cavity through M2 without reflecting and was removed with a reflective filter (OD > 6 at 800 nm) after the cavity. As mirror M2 was 7% transmissive to 400-nm light, and the BBO crystal had 6% losses for each pass through the crystal (reflection and absorption), 82% remained in the cavity after each successive round trip (87% after the first pass). Frequency-doubling and reflective losses within the cavity generated the pulse train with 21% conversion efficiency (with 5.75 mJ of 800-nm input) for the pulse train leaving the cavity. Each frame was normalized in intensity by adjusting the gain at the corresponding CCD and by post-processing to adjust the brightness. Random noise from high gain was compensated for with image processing by smoothing with Speckle filters, as described in the Supplemental Information. The time delay between successive pulses was determined by the cavity length, and measured on a digital oscilloscope. The probe spot was telescoped to a 1.5 mm diameter waist at the sample.

Two separate imaging systems—shadowgraph and dark-field—were used to measure the shock progress in the plane orthogonal to the beam propagation direction. In shadowgraph imaging (Fig. 1c), maps of the amplitude of the light passing through the sample were collected with a narrow depth of focus (~4 μm) within the image plane. For this system, *L*_1_ was a 10X infinity-corrected objective, used to achieve good spatial resolution and image quality, and *L*_2_ had *f* = 1000 mm to expand the field of view. Dark-field imaging (Fig. 1d) created spatial maps showing variations in the refractive index resulting from the shock. The system was constructed using a three-lens imaging system, with *L*_1_ the same 10X objective, *L*_2_ an *f* = 150 mm lens, and *L*_3_ an *f* = 300 mm lens. Phase-to-amplitude conversion between the sample and detectors was achieved by placing a mask in the Fourier plane of the object within the imaging system to block part of the 0^th^-order light that was not deflected significantly from its original wavevector direction after passing through the sample assembly. The mask in this work used a gold rectangle (transmission, T ≈ 0.37 at λ = 400 nm) of 20 μm × 100 μm × 10 nm dimensions on the surface of a 2 mm thick fused-silica double-sided optical flat. As the mask was later found to be damaged, subsequent experiments were performed using a 100-nm-thick, 88-micron diameter round mask, which yielded images that were extremely similar. Using the imaging systems, we resolved features <2 μm in size with a field of view of 130 μm × 170 μm for the shadowgraphs and 330 μm × 440 μm for the dark-field images. The 3 ns duration between frames was the shortest possible in this configuration, as set by the capabilities of the camera.

## Experimental Results

### Shadowgraph imaging

**Figure 2:**
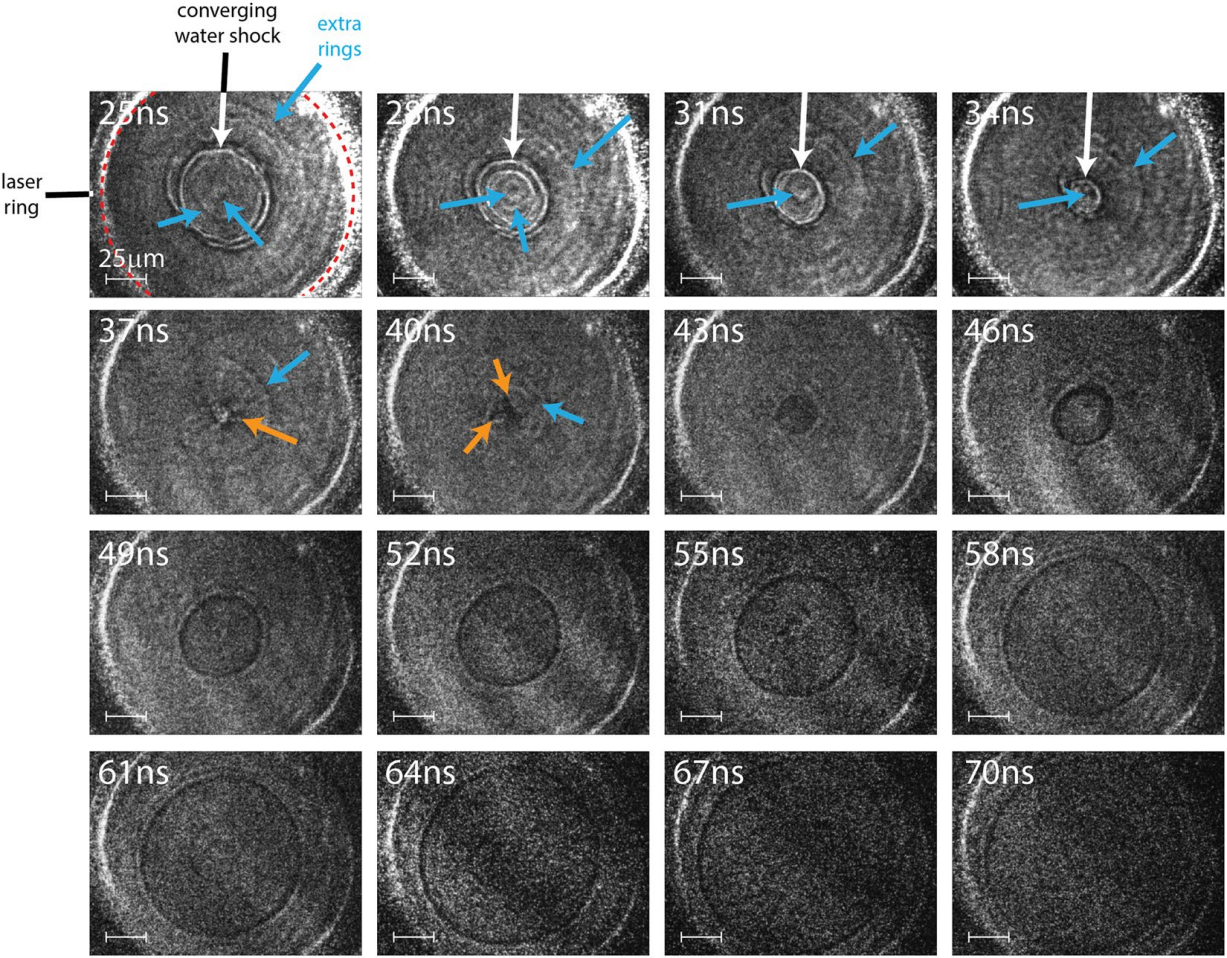
Single-shot multi-frame shadowgraph images showing shock convergence and subsequent divergence in the multi-layered target. The inner edge of the laser excitation line is shown in red in the first frame, white arrows point to the water shock front, blue arrows show additional image features, and orange arrows point to geometric instabilities. The image at 28 ns includes a ghost image due to some light from the 31-nm probe pulse reaching the 28-ns CCD.

Figure 2 shows a sequence of shadowgraph images that demonstrate our single-shot method to image cylindrically propagating shock waves. Image sequences like the one shown here allow us to track non-reproducible events like the geometric instability seen in Fig. 2 and discussed later in this section. Shadowgraph image signal comes from probe light refraction due to refractive-index changes in the material, in this case caused by density variations from the shock wave^5,17^. The imaging light is sensitive to the spatial second derivative of the refractive index, ${\nabla }^{2}n=\frac{{\partial }^{2}n}{\partial {{\boldsymbol{x}}}^{2}}+\frac{{\partial }^{2}n}{\partial {y}^{2}}$, in the object plane^5^. This means that our shadowgraph image sequences are most sensitive to the shock front or other abrupt index changes in the target.

The 16 frames in Fig. 2 are separated by 3 ns intervals, with the 130-fs probe pulse duration setting the integration time for each image. The laser excitation ring, highlighted with red dashed lines in the first frame, produced the white outer ring that is stationary throughout the entire sequence. Frames from 25 ns to 37 ns display concentric rings that get smaller with increasing time, corresponding to the shock front in water converging to the center of the excitation ring. The 40-ns frame shows a dark shape at the center of convergence, with slight geometric distortions, and all subsequent images (43–70 ns) show the rings expanding outward as the shock diverges. We observe imperfect circles from the converging shock front (25–37 ns) and when the shock reaches the focal point at 40 ns. While a geometrically stable converging shock wave would appear as a dark circular spot at the shock focus, Fig. 2 reveals a dark shape that deviates significantly from a perfect circle. This non-circularity upon convergence indicates geometric instability that originates from inhomogeneities in the mode of the drive laser and sample inhomogeneities^18,19^. Other image sequences also show instability, but the precise evolution is different in each shock event. While the converging wave’s geometry distorts as it approaches the focus, the diverging wave maintains a nearly circular structure. This type of geometric instability is characteristic of converging shock waves^20–22^, but its details near the center of convergence have rarely been resolved as they are here, with short temporal and spatial intervals between successive images in our measurements.

Close examination of all frames of the image sequence shows faint concentric rings, shown with blue arrows, in addition to the main ring. These features are most clearly seen in the frames collected at 25 ns, 31 ns and 37 ns, although they are present in every frame. As we will describe later on, tracking these faint image features enables us to follow shock behavior (e.g. substrate shocks, coupled wave interactions, etc.) that would be difficult to discern in single-frame experiments.

### Dark-field imaging

Dark-field imaging enabled us to gather a complementary multi-frame view of shock progression in our multi-layered targets. In dark-field imaging, the signal originates from variation in the optical phase that the probe beam accumulates as it propagates through the transparent target. As the imaging light field propagates through the target, it acquires a phase $\varphi =\frac{2\pi n}{\lambda }\ell$, where *n* is the refractive index, *ℓ* is the thickness of the target in the region with that value of refractive index, and *λ* is the wavelength in air. The cylindrical shock waves change the refractive index along the radial coordinate (distance from the center of convergence) *R*_*S*_, the angular coordinate *θ*, and the target depth *Z*. Consequently, the total accumulated phase is $\varphi ({R}_{s},\theta )={\varphi }_{0}+$ ${\rm{\Delta }}\varphi ({R}_{s},\theta )=\frac{2\pi {n}_{0}}{\lambda }\ell +\frac{2\pi }{\lambda }{\int }_{0}^{\ell }{\rm{\Delta }}n({R}_{s},\theta ,Z)dZ$, where *ϕ*_0_ and *n*_0_ are the average phase and refractive index. Density variation from the shock wave creates local refractive-index changes, Δ*n(R*_*s*_, *θ*, *Z)*, that result in corresponding local phase shifts, Δ*ϕ(R*_*S*_, *θ)*, in the imaging light. The value of Δ*n(R*_*s*_, *θ*, *Z)* at a given position corresponds to the difference in refractive index between that point and the average index for the *R*_*S*_, *θ* plane at that *Z* position.

We convert the object phase pattern into an amplitude pattern that our camera can detect by placing a mask at the focus of *L*_2_ (Fig. 1d), i.e. the Fourier plane of the target. By blocking most of the undeflected light with the gold mask, we cause the light hitting our detector to primarily originate from the deflected, phase-shifted light that was influenced by the shock^23,24^.

The dark-field imaging configuration described in this work is similar to Schlieren imaging, but the differences in its implementation create subtle differences that change its sensitivity to anisotropic changes in a material. As both Schlieren and dark-field imaging use amplitude masks in the Fourier plane of the imaging system to convert the phase variation to amplitude for the images, they both produce signal whose amplitude depends on the difference between the local refractive index and the average total index of refraction. However, Schlieren imaging uses a knife edge to block more than half of the spatial frequencies of the image in the Fourier plane. This means that light that is deflected at an angle θ from the focal plane of the lens is collected based on the direction of its deflection (i.e. the sign of θ). By using a rectangular bar to mask the undeflected light in the Fourier plane of the imaging system, the dark-field images collected in this work include both positive and negative angles of deflection, θ, and excludes only un-deflected light^5,25^.

**Figure 3:**
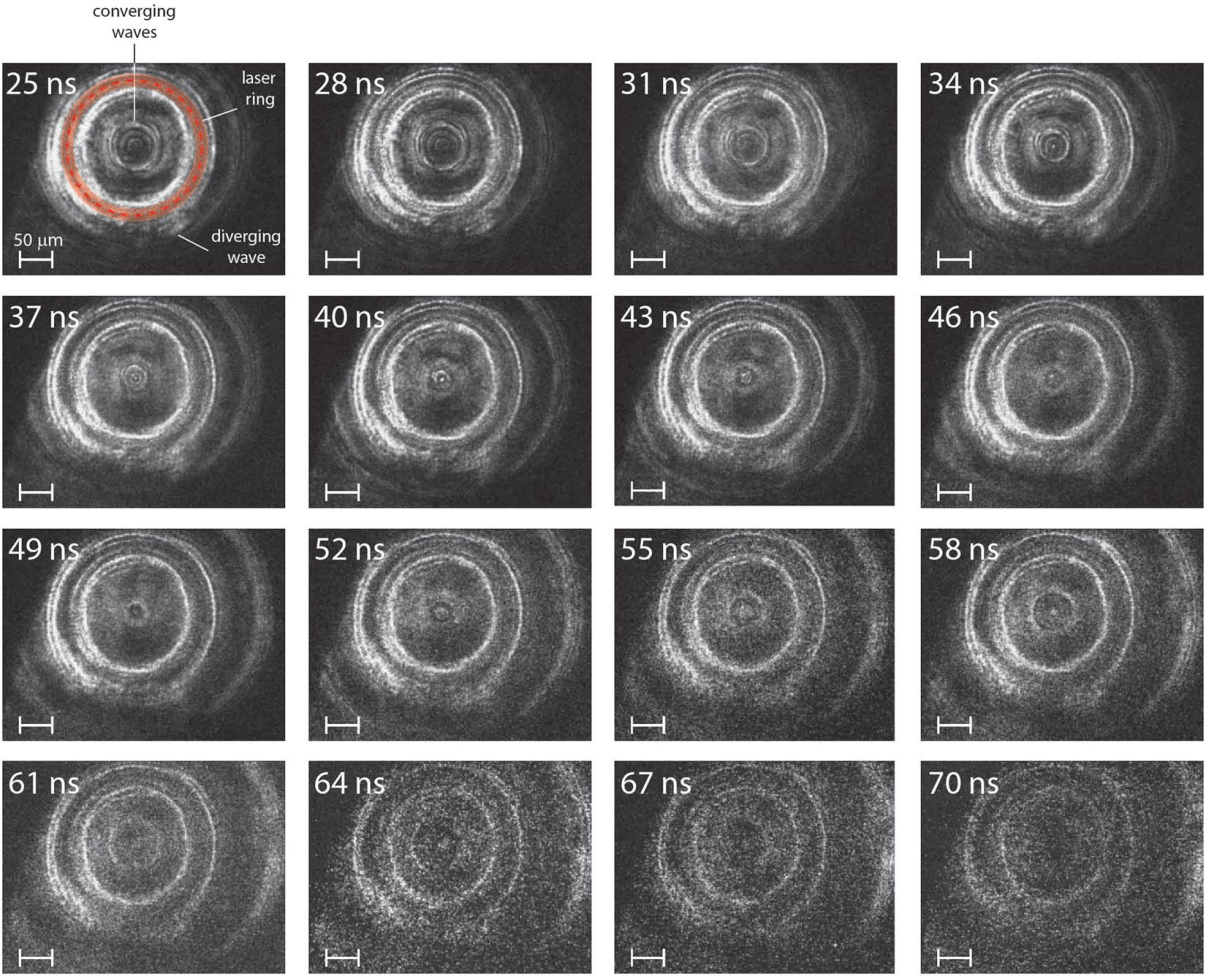
Dark-field image sequence showing shock convergence and subsequent divergence in the multilayered target geometry. The shock was generated with the same drive conditions as in Fig. 2, though the sample is slightly shifted along the *z*-axis, causing the shock ring to have a radius slightly larger than 100 µm. The bubbles from the drive laser excitation ring are indicated by the red line and translucent red band in the first frame.

We show a dark-field image sequence collected with the multi-frame configuration in Fig. 3. The intensity of light at each pixel is proportional to 1 − cos(Δ*ϕ*), revealing regions where there were large phase shifts accumulated through the target. Bright features originate from changes in the refractive index, which are dominated by changes to the density^24^. Signal from bubbles produced by the laser excitation ring are highlighted by a translucent red band and the region that was irradiated by the laser is highlighted by the red dashed line in the first frame (the un-highlighted signals are repeated in each subsequent frame). The images capture a larger field of view (lower magnification) than those in Fig. 2, showing the diverging shock outside the laser excitation ring in addition to the converging shock within.

Consistent with the shadowgraph sequence, we observe multiple concentric rings inside the laser ring for each image across the entire 25–70 ns period captured in the sequence. Both image sequences enable us to track multiple rings, but shot-to-shot experimental variation prevents precise correspondence between different sequences. While multiple rings are quite clear for the waves inside the laser excitation ring, they are not discernable for the lower amplitude diverging wave outside the excitation ring. The unusual behavior of these extra rings suggests that their cause is strongly dependent on the shock amplitude, which is highest for the converging wave inside the laser excitation ring. Multi-wave structure in shocks can be caused by phase transitions in the shocked material^26^, but as discussed further below, in this case it is more likely a result of only partial confinement of the shock in the water layer.

## Simulation Results

The simulations were conducted using the CTH shock physics code, developed at Sandia National Laboratories^27^, with setup parameters matching those from the experiment as closely as possible. We used the simulated density maps to calculate the refractive index variations in the target and the phase shifts imparted to our imaging light in order to model the images we observed. To understand the origin of the additional rings we observed in both image sequences, we extended previous simulations^6^ to include all five target layers: Air, Sapphire, Water, Sapphire, Air. Due to experimental uncertainties in the water layer thickness (10–20 μm) and quantitative differences between the real and simulated shocked sample assemblies (for example, the simulation does not account for shock anisotropy in sapphire), the simulations provide a qualitative picture of shock propagation in the targets. Pressure-dependent values of the water photoelastic constants^28–30^ were used to calculate the shock-induced refractive index changes in the water sample layer.

**Figure 4:**
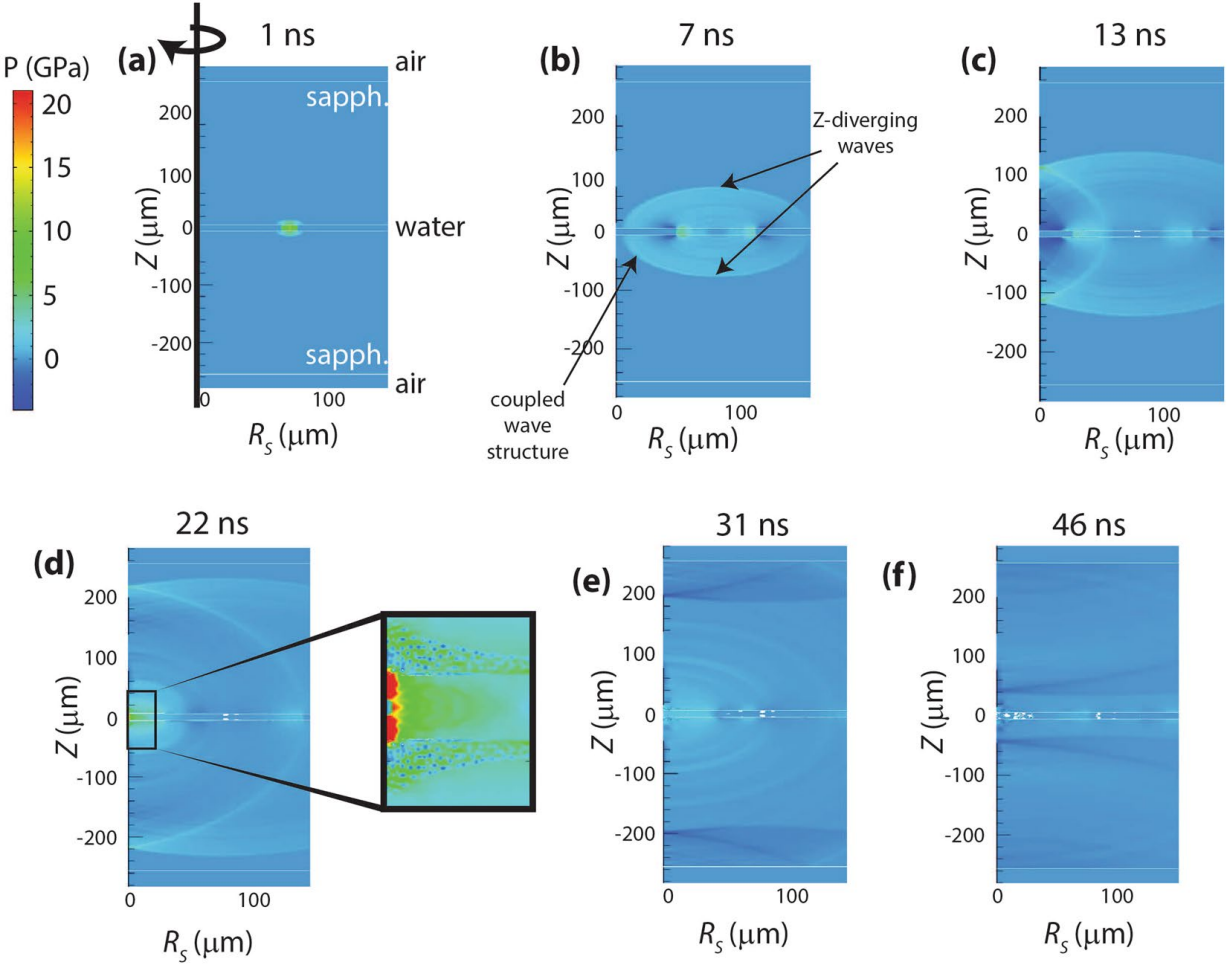
Axisymmetric view of the shock simulated across the target geometry, displaying pressure changes along the depth and radius. The time steps show the shock convergence and subsequent divergence, and indicate the *Z*-diverging and coupled-wave shock components.

Figure 4 shows an axisymmetric view of the samples, with the color-code indicating the pressure at each radius *R*_*S*_ and depth *Z* in the target. Figure 4a shows the result of the initial heating of the irradiated water region, causing high pressure and thermal expansion which launches shock responses in all directions. Expansion in the vertical (*Z*) direction launches shocks that propagate and diverge in the sapphire substrates. These are clearly observed in Fig. 4b–d. Some components of these waves reach the sapphire-air interfaces (see Fig. 4e) and later (at times *t* > 50 ns) return to the water layer, but by then they have diverged and have negligible pressure. Thermal expansion of the irradiated water region in the sample plane launches the water shock that is cylindrically focused and reaches the highest pressure at the center of convergence as seen in Fig. 4b–d. (The diverging water shock is also apparent in Fig. 4b–d, moving away from the focal region). We will refer to this quasi-confined water wave as the primary shock. The inset at *t* = 22 ns (Fig. 4d) shows the highest pressure reached in the simulation, 21 GPa, in the water layer at the center (observed in our shadowgraph images at 40 ns).

Because the water-sapphire impedance mismatch leads to only partial confinement of shock pressure within the water layer, during its convergence the primary shock transmits part of its mechanical energy into the substrates, launching the hemi-torroidal shock waves in sapphire that converge along *R*_*S*_ while diverging in *Z*.

The primary water shock also extends through the interfaces and into the nearby regions of the substrates as it propagates toward the focus. The shock speed is faster in the substrates, which causes the leading substrate shocks to extend back into the sample layer, creating a pre-shock for the primary wave. This coupled-wave structure consists of the primary water shock, an oblique sapphire shock in both of the nearby substrates, and an oblique water pre-shock.

The multi-wave interactions in the coupled-wave structure are introduced by reflections and transmissions at the interfaces and shift primary water shock’s pressure-particle velocity (*P-u*_*p*_) state away from the principal Hugoniot. While the standard principal Hugoniot equation defines the constitutive relations for shocked materials, it is only strictly valid for a uniaxial shock wave travelling in a material that is initially at rest under ambient conditions^31^. In our system, both the converging geometry and the interface effects introduce perturbations to the principal Hugoniot relations among the material properties in the shocked material (i.e. *P*, temperature *T*, shock velocity *U*_*s*_, *u*_*p*_, density *ρ*), causing deviations from expectations for a planar shock in a single-component system.

**Figure 5:**
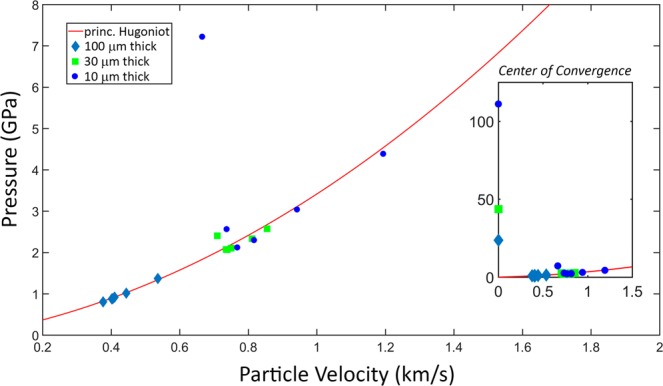
Plot showing the particle velocity versus pressure for samples of three thicknesses against that of the principal Hugoniot. The points in each case are taken from the simulation results at radial locations indicated in Table 1 (*R*_*s*_ = 0, 8, 16, 24, 32, 40, 48 μm). The inset shows the results at the center of convergence, where the pressures increase sharply but the particle velocities from shock components propagating in opposite directions cancel, yielding no net particle velocity. The effect is a strong deviation from the Hugoniot at the focus. The outlying point at ~7 GPa is almost at the center of convergence (8 μm away).

To investigate how the coupled-wave structure caused the *P* and *u*_*p*_ values to deviate from the principal Hugoniot, we compared the simulated *P* and *u*_*p*_ values for the shock front at selected radial positions during shock convergence. We repeated the simulations from Fig. 4 for sample thicknesses of 30 μm and 100 μm to examine how an increased volume of sample that is located further from an interface changes the relationship between *P* and *u*_*p*_. The *P*-*u*_*p*_ states reached at seven different radial points upon convergence are displayed in Fig. 5 and indicated in Table 1 for all three sample thicknesses. Figure 5 includes a plot of the principal Hugoniot, for comparison to the states reached by the waveguide geometry.

**Table 1:** Comparison of values to demonstrate how the *P-u*_*p*_ values at the shock front deviate from the principal Hugoniot at seven radial positions as the shock converges, for waveguides with 10 μm, 30 μm and 100 μm water layer thicknesses.

Radius [μm]	10 μm Waveguide			30 μm Waveguide			100 μm Waveguide		
	*u*_*p*_ [km/s]	*P* [GPa]	dev^(a)^	*u*_*p*_ [km/s]	*P* [GPa]	dev^(a)^	*u*_*p*_ [km/s]	*P* [GPa]	dev^(a)^
48	1.2	4.3	1.8%	0.86	2.6	1.8%	0.38	0.80	1.7%
40	0.94	3.0	2.3%	0.81	2.4	4.6%	0.40	0.88	0.42%
32	0.74	2.6	10%	0.75	2.1	2.8%	0.40	0.88	0.23%
24	0.82	2.3	5.3%	0.74	2.1	0.74%	0.41	0.91	1.6%
16	0.76	2.1	5.2%	0.74	2.3	1.3%	0.44	1.0	2.0%
8	0.67	2.7	57%	0.71	2.4	11%	0.54	1.4	3.1%
0	0.00	110	—	0.00	44	—	0.00	24	—

^a^Percent deviation from the principal Hugoniot.

The principal Hugoniot defines the jump conditions for a single uniaxial shock wave^32^, using the Rankine-Hugoniot conditions, which describe conservation of momentum, energy and mass for a uniaxial shock^1,31^. At the focus, all converging shocks reach a thermodynamic state far from the principal Hugoniot, as the net particle velocity approaches zero while the pressure approaches a singularity as the counter-propagating shocks around the ring collide^33–36^. The thinnest sample layers reached the highest pressures, indicating that the interface effects enhance the pressure of the system at all positions upon convergence, even with only partial confinement.

Figure 5 indicates that the thinner samples have *P*-*u*_*p*_ states that deviate most from the principal Hugoniot. A more detailed view of the behavior may be seen in Table 1, which includes the simulated values for *P* and *u*_*p*_ that are plotted in Fig. 5 with the corresponding radial positions. For all *P*-*u*_*p*_ points given in Table 1, the percent deviation was computed by comparison to the nearest point (minimum distance) along the principal Hugoniot. The results show that for the 100-μm liquid layer, there is a very gradual increase in pressure and particle velocity as the shock propagates toward the focus, with a sharp pressure rise indicated only at the focus itself. That indicates that the increase in shock amplitude due to focusing slightly outweighs the decrease in amplitude due to damping in the liquid layer or other dissipative effects. In the 30-μm waveguide, there is a gradual decrease in pressure and particle velocity at first (from 48 μm to 32 μm radius), then a gradual increase until the sharp rise at the focus. Since the results from the 100-μm waveguide already demonstrate that dissipation in (essentially) bulk water does not outweigh focusing, it is clear that interface effects play a role, presumably through coupling of the wave in the liquid layer to the substrates around it. Interface effects are strongest in the 10-μm waveguide, in which the pressure and particle velocity decrease considerably from 48 μm all the way to 16 μm, and only begin to show an increase between 16 μm and 8 μm before the sharp rise at the focus. This illustrates that the interface effects can result in lower shock amplitudes on the way toward convergence, even while the very high pressure reached in the 10-μm waveguide right at the focus indicates that substrate and interface effects can enhance the pressure there.

It is notable that in all three samples, the deviations from the Hugoniot are relatively small until the focus is approached closely. Before then, even in the 10-μm waveguide, the deviation reaches at most 10%, and among our tabulated values that occurs only once, with no other deviation significantly greater than 5%. This suggests that away from the focus, we can obtain reasonable estimates of the shock pressure in the liquid layer from the shock velocities determined through optical imaging.

## Discussion

We now consider the manner in which the simulated shock structure gives rise to distinct features in imaging measurements, and we compare the results to the experimentally measured features including multiple rings. The coupled-wave structure extends through the water layer and into the nearby substrate regions, but water’s far larger photoelastic constant means that the images we observe are primarily due to pressure-induced refractive index changes in the water. Using the hydrodynamically simulated densities and the reported pressure-dependent photoelastic constants^28,29,37^, we calculated the predicted refractive index values in each layer. When the primary water wave reaches the center of convergence, the water index varies by Δ*n*_*water*_ ~ 0.5, while sapphire’s lower shock pressures and photoelastic constants cause each substrate in the immediate vicinity of the water layer to undergo a maximum change of Δ*n*_*sapph*_ ~ 0.03. This suggests that the additional rings in our images originate from the water component of the coupled-wave structure. Using our predicted change in the refractive indexes, we demonstrate a qualitative comparison of the phase and amplitude contributions that the shock would impart onto the image. The discussion below only provides a qualitative description of the image signal, as our models ignore other contributions (i.e. those from scattering, absorption, aberrations, etc.).

The imaging methods capture different aspects of the shock dynamics in our targets. Shadowgraph imaging is sensitive to abrupt and large variations in the refractive index, showing the shock front but not the entire wave^5^. In contrast, dark-field images can capture lower-amplitude and more gradual density changes in the shock because of the signal’s accumulated Δ*ϕ*-dependence^5,17,38,39^. These different signal dependences enable a clear view of the leading oblique waves with dark-field imaging, while resolving the position of the primary water shock front in shadowgraph images.

**Figure 6:**
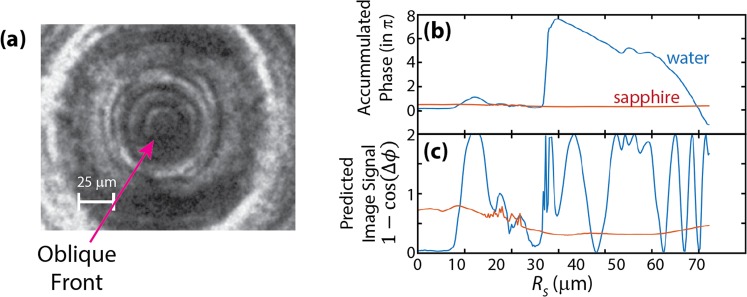
(**a**) Dark-field image collected 25 ns after the drive pulse, compared to the simulated values (from the corresponding 13-ns delay) showing (**b**) the accumulated phase the light acquires through the sample in units of $\frac{\Delta \varphi }{\pi }$, and (**c**) the predicted signal $1-\,\cos ({\rm{\Delta }}\varphi )$ that would arise from the separate water and sapphire signals, illustrating both the sensitivity and the over-rotation.

Dark-field images produce signal from the accumulated phase change experienced by the light passing through the target, making its dependence on refractive index $1-\,\cos ({\rm{\Delta }}\varphi )=\,1-\,\cos (\frac{2\pi {\rm{\Delta }}n}{\lambda }\ell )$ for each region $\ell$ over which there is variation in ${\rm{\Delta }}n$. Standard dark-field images show position-dependent intensity $I\propto {\rm{\Delta }}{\varphi }^{2}$ for small phase shifts, following the small-angle approximation. In our case, the accumulated phase cycles repeatedly over intervals of 2π. Using our calculated refractive index values from the simulation for a 13 ns delay (Fig. 4c), we show the accumulated phase through the water (blue) and sapphire (red) layers of the target in Fig. 6b. We associate this simulated delay approximately with the experimental image recorded at 25 ns delay based on the difference between simulated and experimentally measured shock speeds. The plots in Fig. 6b shows that the dark-field image includes phase shifts of up to ~8π through the water layer and ~0.2π through the sapphire, the latter varying only slightly with position as it includes the wave components of the hemispherical diverging wave that has spread through the Z-dimension within the sapphire. Firstly, this means that the imaging signal primarily originates from the water layer, though there is a small contribution from each sapphire substrate, as seen in Fig. 6c. Additionally, the sinusoidal relationship between image signal and phase causes the water layer to over-rotate the phase shift, creating artificial features in the images from the phase cycling, as seen at 35 μm, 45 μm and >60 μm in Fig. 6c. While phase cycling generates extra features, this technique’s high Δ*n*-sensitivity causes density variation within the profile of the coupled-wave structure to be well resolved in the image signal. The predicted image signal shown in Fig. 6c shows that the leading oblique wave (the pre-shock in water) should be well resolved in our dark-field images, and the primary water shock should give rise to a distinct ring at the shock front followed by multiple additional rings as the phase shifts from trailing edge of the shock descend through various integer multiples of π. The comparison between the simulation and the data in Fig. 6a is not quantitative, but the qualitative features appear as expected. While the dark-field images show extra features due to over-rotation, their sensitivity to the cumulative phase shift gives detail in the structure of the oblique shock front, providing information complementary to that from the shadowgraph images.

**Figure 7:**
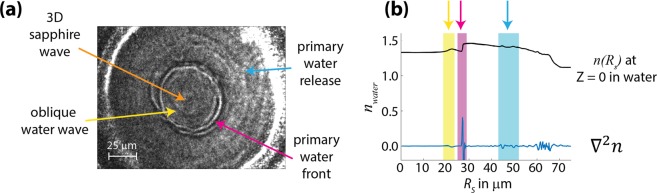
(**a**) Assignments showing the origin of each ring in the shadowgraph image from 25 ns. This is compared to (**b**) plot showing the simulated refractive index and spatial second derivative (Laplacian) of the refractive index for the center of the water layer as a function of radius. The simulation is from 13 ns, as the simulated shock velocities were faster than those observed.

Our shadowgraph images show significantly fewer concentric rings, as illustrated in Fig. 7a in which the image shows one clear primary ring between two faint ones. Figure 7b shows the predicted refractive index in the center of the water layer (i.e. *Z* = 0) and its spatial second derivative, ${\nabla }^{2}n$, giving assignments for each ring in our images. We calculated the Laplacian of the full image for the four microns of the water layer centered at *Z* = 0, corresponding to the depth of focus of our imaging system, and we present a radial slice from the resulting symmetric derivative map in Fig. 6c. At *t* = 25 ns in our experiment (corresponding to 13 ns in the simulation), we see one primary ring, with three additional faint rings. Comparison between the simulations and experimental images clearly indicates that the single darkest ring corresponds to the shock front of the primary water wave, which has the highest ${\nabla }^{2}n$ value. Within the image sequences in Fig. 2, we can resolve the progression of two additional waves, one preceding and one following the primary shock front. From our simulations, we interpret that both the leading (yellow) and trailing (blue) faint rings originate from the water components of the coupled-wave structure. We attribute the leading faint ring to the oblique water shock, which both the image and simulation (Fig. 6b) show precedes the most intense signal from the primary water wave.

At the trailing edge of the coupled-wave structure, the simulations predict two types of pressure release: a release from the primary water wave, and a subsequent release from the oblique waves. In both cases, the magnitudes of the density changes should be relatively large (largest for the primary water wave), but the release profiles should be relatively slow, causing their second derivatives to be relatively small. This translates to faint signals in our shadowgraph images, as shown by the blue arrow in Fig. 7a. Our predictions show that the primary water release (shown in blue) trails the primary feature in subsequent images throughout Fig. 2.

The feature at the center of the shadowgraph image in Fig. 7a does not originate from the water layer, according to the simulation and consistent with our expectation that it is too early for any wave component in the water to have reached the focus. This indicates that the feature arises from the sapphire layers. Two factors cause the wave to be visible despite the low photoelastic constant of sapphire. In Fig. 4, it is evident that components of the diverging waves in the substrates reach the radial center (*R*_*S*_ = 0) first at *Z* = 0 (this has almost occurred at 7 ns, in Fig. 4b) and then at successfully greater distances from *Z* = 0. At 13 ns, it is occurring at *Z* ≈ ±110 μm. The resulting pressure in that region results in a change in the sapphire refractive index of roughly 0.08, and this is sufficient to be detectable in our images. Even at long times, diverging wave components continue to reach *R*_*S*_ = 0 after reflections from the sapphire-air interfaces. Therefore our images show a central feature that appears stationary and that changes in intensity between frames.

**Figure 8:**
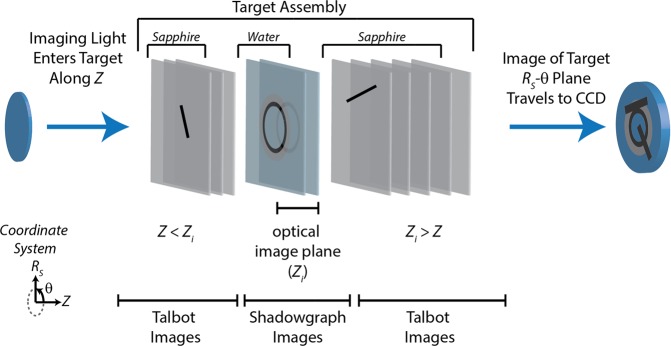
Illustration of signal contributions in our shadowgraph imaging system from different regions along the *Z*-axis of our target assembly. Shadowgraph and Talbot contributions from different target layers are overlaid in the images.

The *Z*-variation of the 3D sapphire wave components that converge at the focus highlights a further imaging consideration that is illustrated in Fig. 8. The ~4-μm depth of focus along the *Z*-axis, set by our 10X objective, places the substrates and air outside of the image plane (*Z*_*i*_). This means that some of the water layer produces a shadowgraph image at the detector, while the remainder of the target generates superimposed phase-sensitive and *Z*-sensitive Talbot images^40^. Changes to the index of refraction in each *R*_*S*_*-θ* plane along *Z* (through all layers) can generate additional features that originate from overlapping Talbot image components (Fig. 8). These effects are small for most of the target, but at the center of convergence, the sapphire reaches a sufficiently high pressures to produce measurable Talbot image signals.

Qualitative comparison of our dark-field and shadowgraph image sequences shows that the different image sources provide complementary information about the shock behavior. However, small fluctuations in shock behavior between laser shots prevent detailed comparisons between the features produced from different shock events. While the converging shock geometry amplifies the shock pressure, it also amplifies the ~5% energy fluctuations and small spatial variations in our drive pulse, as well as inhomogeneities in our sample, creating geometric instabilities^20^. This shot-to-shot variation prevented us from tracking the temporal progression of all the wave components when we used a single-frame camera to record just one image during each shock event, so that images at different time delays were recorded from different events^6^. With multiframe imaging during a single shock event, individual frames within a single sequence may be compared quantitatively. The ability to track irreproducible phenomena also enables us to monitor the temporal progression of shock-induced material decomposition and fracture, deformation of shock geometries due to propagation into different materials within heterogeneous targets, and other behavior that varies substantially from shot to shot.

The combination of the dark-field and shadowgraph imaging sequences and comparison to simulations have given us a new and more detailed view of shock waves in our quasi-confined geometry. In both imaging methods, the extra rings in our images could be interpreted because the single-shot sequences enabled us to track the motion of each ring from frame to frame. Our dark-field images gave a set of rings in each image that showed the oblique front and release, while over-rotation created extra features within the primary shock. This information was complemented by the shadowgraph image sequence which contained mostly information about the primary water wave. The different sensitivities of the two methods to different features of the shock enabled us to interpret all of the main features that we observe in terms of features that were revealed in numerical simulations. The interpretation was facilitated by the low photoelastic constant of the sapphire substrates, and in general this feature is highly desirable for imaging of shock waves in a multilayer target like ours.

The coupled wave geometry enhances the pressure reached at the shock focus while it introduces complexity in determining the *P-u*_*p*_*-U*_*s*_ state of the system. Previously, we inferred the average shock pressure with the 10 μm thick samples by inputting the measured shock velocity between frames to the principal Hugoniot equation of state^6,12^. The simulations in this work demonstrate that thinner samples in the waveguide geometry reach higher pressures at the center of convergence. Our results indicate that the deviations can be minimized by increasing the sample layer thickness, and that a reasonable balance is possible (in our case at around 30 μm) to reach substantial pressures that can be determined with sufficient accuracy from shock velocity measurements.

Numerical modeling revealed key elements of the coupled wave structure in our target geometry. We have conducted additional modeling of targets with materials of different impedances. For the study of solid sample materials, inverting the impedance mismatch to sandwich a high-impedance sample layer between two low-impedance substrates can simplify the coupled-wave structure since in that case the fastest wave speed is in the sample layer. In separate experiments, we have used dye-doped, low-impedance polymers for absorption of pump light and generation of shocks. Modeling and experiments have indicated that such layers sandwiching a higher-impedance silica glass layer were effective in launching a shock within the glass layer whose propagation and focusing within that layer were almost completely unaffected by the surrounding polymer layers, resulting in relatively simple wave characteristics and pressures that could be determined reliably from shock velocities. This approach is not possible for liquid samples, but as indicated above, their shock pressures can be determined with adequate accuracy if the thickness is well chosen.

## Conclusions

This paper presents the development of single-shot multi-frame imaging that has extended our understanding of a tabletop platform for generation and measurement of quasi-2D converging shock waves^6^. We used a frequency-doubling Fabry-Perot cavity to generate a train of femtosecond pulses for illumination of all the frames recorded by a high-frame-rate camera. The experimental scheme was demonstrated for shadowgraph and dark-field imaging. In our multi-layered sapphire-water-sapphire target geometry, the image sequences all showed multiple concentric rings originating from the primary shock in the water sample layer and from additional shock structure. The coupled-wave structure caused by the sapphire-water interfaces resulted in additional oblique waves in the water that created additional rings in our image sequences. Numerical simulations revealed the additional shock components and allowed prediction of the image features that agreed qualitatively with our measurements. Our imaging technique and understanding of the coupled-wave structure can inform future sample design and material studies in this tabletop experimental geometry. The single-shot imaging presented here is useful for understanding shock behavior that shows temporally complex dynamics with shot-to-shot variation, and the approach is compatible with extremely small sample volumes. For example, in work to be reported separately, we have used the method to examine shock-induced decomposition of energetic crystallites of ~10 μm dimensions embedded in polymer hosts, combining imaging with spectroscopic measurements to characterize structural and chemical decomposition kinetics^41^. Variations in size and shape from one crystallite to the next lead to differences in the details of the responses that would make the kinetics extremely difficult to extract from only one image per shock event, using measurements on different crystallites to record the responses at different time delays following shock generation. Sequences of 16 images allow each event to be monitored sufficiently for the kinetics to be discerned. Our approach is also compatible with time-resolved X-ray diffraction and scattering, similar to recent measurements in which the shock propagation direction was approximately perpendicular to the X-ray propagation direction^42^ but with far lower pump laser intensities and sample volumes.

## Supplementary information

Supplemental Information

## Data Availability

The primary datasets described in this work are included in the paper and Supplemental Information. Additional datasets were generated for statistics and are available from the corresponding author on reasonable request.
