# Supplementary material for: Single-Shot Multi-Frame Imaging of Cylindrical Shock Waves in a Multi-Layered Assembly
Source: Sci Rep. 2019 Mar 6;9:3689. doi: 10.1038/s41598-019-40037-3 (PMC6403302; doi:10.1038/s41598-019-40037-3)
Supplement: Supplementary file 1 — Supplemental Information [file 41598_2019_40037_MOESM1_ESM.pdf]

Single-Shot Multi-Frame Imaging of Cylindrical Shock Waves in a Multi-Layered Assembly

Leora Dresselhaus-Cooper*, Joshua E. Gorfain, Chris T. Key, Benjamin K. Ofori-Okai, Suzanne J. Ali, Dmitro J. Martynowych, Arianna Gleason, Steven Kooi, Keith A. Nelson

Supplemental Information

Image Processing Description

The image sequences shown in this work were processed using the white balance, contrast and noise enhancement features from the ImageJ software package. The TIF images collected from the camera were first manually white-balanced, then noise filters were applied. We used the built-in despeckle to remove the random pixel noise produced by gain in the detectors. Figure S-1 shows the entire shadowgraph image sequence before (a) and after (b) the despeckle filter was applied. The image processing did not change the visible or resolvable image features, in all cases.

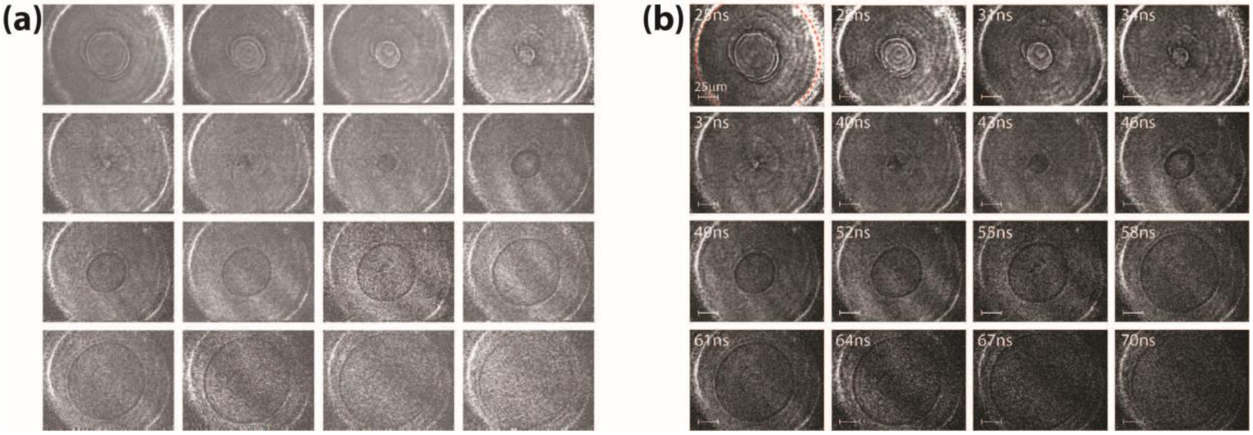

Figure S-1. Image sequence from Figure 1 in the main paper (a) after the white-balance but before the contrast enhancement and subsequent despeckle filtering, and (b) processed.

Description of Simulations

Our simulations included all five layers (air-sapphire-water-sapphire-air) in the target. No assumptions were made regarding local boundary conditions, allowing interface locations to vary. Computational domain boundaries were placed far enough from the experimental region of interest that artificial boundary wave interactions were avoided during the time scale of the simulated experiments.

The simulations did not treat laser light-matter interactions explicitly. We assumed that 90% of the energy from the drive laser pulse was transferred into the water (based on previously reported sample transmission[1]; no light absorption in other target layers was considered) with spatial and temporal profiles based on the experimental parameters, i.e. 150 μm laser ring inner diameter, 8 μm thick laser ring line width, and 100 ps Gaussian temporal profile. Rapid heating of the

irradiated region induces a compressive shock, which is alleviated by a rapid expansion that initiates bulk shocks in the target (and by the subsequent shock dynamics). The laser-induced shock in the simulations were symmetric about the plane of the water layer, such that the shock pressures in the figures are symmetric above and below the $R_S = 0$ plane (centered in the middle of the water layer).

Simulations for the wave dynamics were performed using the CTH shock physics code developed by Sandia National Laboratories.[2] A Mie-Grüneisen equation of state with the Hugoniot as reference was used to describe the hydrodynamic and thermodynamic behavior of the water sample. The principal Hugoniot was determined from a quadratic fit to shock velocity-particle velocity data with parameters c_0 = 1.48 km/s, s_1 = 1.984, s_2 = -0.143, reference density ρ_0 = 0.998 g/cm^3, Grüneisen parameter, Γ_0 = 0.48, and specific heat C_V = 3.69 J/g/K.[3] Sesame tabular equation of state and elastic-perfectly plastic strength models described the behavior of the sapphire substrates. The outer air layers were also modeled with a Sesame equation of state. The computations were performed in an axisymmetric geometry, using a uniform mesh size of 0.25 μm throughout the region of interest.

Details of the Pre-Shock

A detailed view of the pre-shock is shown in Figure S-2, which shows three time slices with a logarithmic pressure scale from our simulated results as the primary water shock converges. This shows that the pre-shock develops and increases in intensity as the primary water wave converges, with a highly non-uniform structure across the *Z*-axis. At 1 ns, as the shocks begin to travel, no pre-shock is evident in the water layer. As the water and sapphire shocks progress, the pre-shock begins to form, as seen at 7 ns. In Figure S-2b, we see a significant pre-shock has formed in the water. The surface component of the initial 3D sapphire wave creates a weak oblique water wave of 10^{-3} GPa pressure, as the sapphire component of the coupled-wave structure begins to move ahead of the primary water wave. At this time, the oblique sapphire waves and primary water shock have not separated enough to induce an additional oblique water wave in the coupled-wave structure. Over the subsequent 9 ns, the oblique sapphire wave moves well ahead of the primary water wave and leaks further energy back into the water layer. By 16 ns, the secondary oblique water wave has a steeply angled front, making it nonuniform along the *Z*-axis, and includes a range of 0.01-1 GPa in pressure.

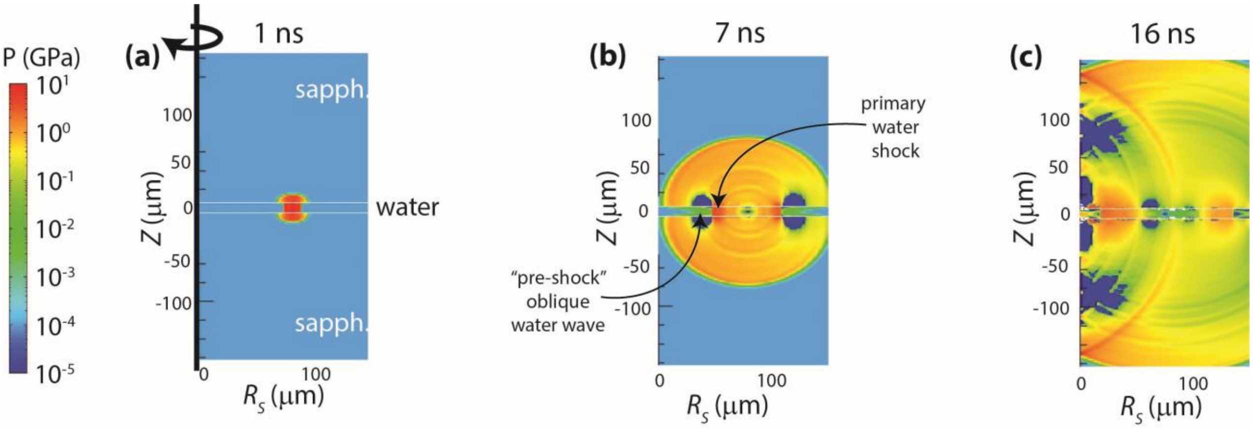

Figure S-2. Simulated results showing a logarithmic pressure scale with a 10-GPa maximum to emphasize the low-pressure disturbances in the water layer.

Through its entire propagation, the pre-shock reaches a maximum pressure of only ~1 GPa which occurs at the center of convergence. During its propagation, the pre-shock shows a pressure between 0.01 and 0.5 GPa, whereas the sapphire waves are ~7 GPa upon convergence while the primary water shock stays at approximately 10 GPa. At almost all times, the pre-shock has at least an order of magnitude lower pressure than the primary water wave.

References

[1] T. Pezeril, G. Saini, D. Veysset, S. Kooi, P. Fidkowski, R. Radovitzky, and K.A. Nelson, Phys. Rev. Lett. **106**, 214503 (2011).

[2] J.M. McGlaun and S.L. Thompson, Int. J. Impact Eng. **10**, 351 (1990).

[3] E.S. Hertel and G.I. Kerley, *CTH Reference Manual: The Equation of State Package.* (Albuquerque, NM, 1998).
